# Supplementary material for: Describing the status of reproductive ageing simply and precisely: A reproductive ageing score based on three questions and validated with hormone levels
Source: PLoS One. 2020 Jun 30;15(6):e0235478. doi: 10.1371/journal.pone.0235478 (PMC7326235; doi:10.1371/journal.pone.0235478)
Supplement: S1 Appendix — (DOCX) [file pone.0235478.s001.docx]

# Supporting Information

## 1. Hormone measurements for validation

We determined FSH and 17β-estradiol in serum at the Core Facility for Metabolomics at the University of Bergen, Norway ([www.uib.no/metabolomics](http://www.uib.no/metabolomics)), using enzyme-linked immunosorbent assays provided by Demeditec Diagnostics, (Germany) for FSH and liquid chromatography - tandem mass spectrometry for 17β-estradiol. All pipetting steps were executed fully automated on a liquid-handling robot from Hamilton Robotics using the protocols 4Elisa and G8Way. For FSH the between day coefficient of variation was 7.0% and accuracy was 106% while the corresponding values for 17β-estradiol ranged between 1.7 – 7.8% and 100 – 105%, respectively. To visualize performance of the reproductive ageing score (RAS), we plotted the RAS against three commonly used categories of menopausal status, defined by hormonal measurements as nonmenopausal with FSH ≤20IU/L and 17β-estradiol ≥147pmol/L; perimenopausal with FSH from 20IU/L to 80IU/L and 17β-estradiol from 73pmol/L to 147pmol/L; and postmenopausal with FSH ≥80IU/L and 17β-estradiol ≤73pmol/L.

S1 Fig. Boxplot of the reproductive ageing score by commonly used categories, defined by hormonal measurements; The median is displayed as horizontal line within the boxes. The lower and upper hinges correspond to the 25th and 75th percentile. The upper and lower whiskers extend from the hinge to the largest, respectively smallest value, yet no further than 1.5 interquartile ranges. Data beyond the end of the whiskers are plotted individually.

##
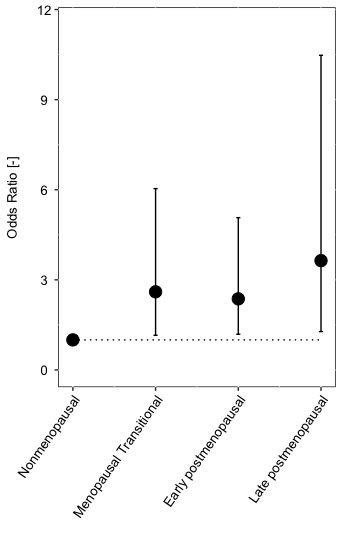
2. Practical example:

**New-onset asthma and menopause**

To demonstrate the utility of the reproductive aging score, we re-evaluated the association between menopause and new-onset asthma, as previously published by our group, using 2085 women from the RHINE study (Triebner et al 2015, J Allergy Clin Immunol). The dichotomous outcome in the original analysis was new-onset asthma. The predictor variable was menopausal status (nonmenopausal, menopausal transition, early postmenopause and late postmenopause). The results show that women in the menopausal transition and postmenopausal women have a two to three times higher risk of developing new-onset asthma (Figure 1). As a comparison we performed the analysis on the exact same data, with the exact same adjustments, using the reproductive aging score instead of the categorical variable. The results continuously cover the whole range of reproductive aging (Fig 2). It is clearly recognizable that the probability of developing new-onset asthma is higher for women who score higher. In addition, a continuous development can be observed. The seamless increments of the score allow estimations of when and how steeply the probability increases. Focusing on the results of a score between 0.90 and 1.00, Fig 3 further indicates that the association is not linear, as this probability eventually decreases again. Such an association, explaining the large confidence interval for the late postmenopausal group, is only demonstrable if the predictor variable is continuous.

S2 Fig. Odds ratio of new-onset asthma according to change in menopausal status (n = 2085) incl. 95% confidence interval.

**
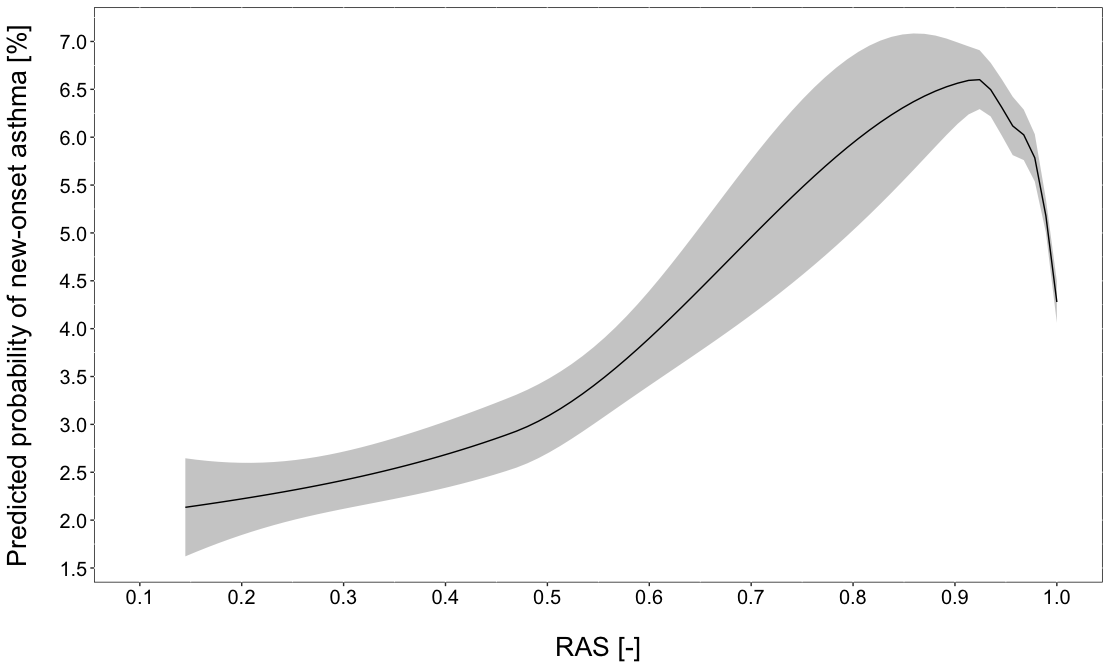
**

**S3 Fig. Probability of new-onset asthma by reproductive aging score for 2085 women, incl. 95% confidence band**, calculated using t-based locally estimated scatterplot smoothing.

## 3. Code for adjusting the datasets

### Reproductive aging score ###

### Kai Triebner ###

### University of Bergen ###

## load development data

df.development <- data.frame(df1)

## drop overlap

df.development <- subset(df.development, df.development$ecrhs==0)

## exclude women answering "No, they have never been regular." to the question: "Do you have regular menstruations?"

df.development <- subset(df.development, df.development$reg.mens=="Yes" |

df.development$reg.mens=="No, they have been irregular for a few months" |

df.development$reg.mens=="No, my periods have stopped")

df.development <- subset(df.development, df.development$pregnant=="No")

df.development <- subset(df.development, df.development$breastfeeding=="No")

df.development <- subset(df.development, df.development$current.contraception=="No")

df.development <- subset(df.development, df.development$current.hrt=="No")

#optional age modifier

df.development$a20<-0

df.development$a20<-ifelse(df.development$ovaries=="yes, one ovary",1,0)

df.development$a20<-ifelse(is.na(df.development$ovaries),0,df.development$a20)

df.development$a21<-ifelse(df.development$smoke=="current smoker",2,0)

#create table 1

table_mu_periods <- na.omit(data.frame(table(rhine$reg.mens,rhine$periods)))

table_mu_periods$sum <- table_mu_periods[,1]+table_mu_periods[,2]+table_mu_periods[,3]

colnames(table_mu_periods) <- c("Regular", "Irregular", "Amenorrhea", "Sum")

table_mu_periods$p.periods <- table_mu_periods$Regular/table_mu_periods$Sum

#create table 2

table_mu_age <- na.omit(data.frame(table(rhine$age,rhine$reg.mens)))

table_mu_age$sum <- table_mu_age[,1]+table_mu_age[,2]+table_mu_age[,3]

colnames(table_mu_age) <- c("Regular", "Irregular", "Amenorrhea", "Sum")

table_mu_age$p.age <- table_mu_age$Amenorrhea/table_mu_age$Sum

The least square approximation for the current article has been carried out with the open-source Maxima CAS software, which can be downloaded here: <https://sourceforge.net/projects/maxima/files/>. Maxima CAS is a specialized computer algebra system that yields high precision numerical results by using exact fractions, arbitrary-precision integers and variable-precision floating-point numbers.

To replicate the current calculations, we recommend using Maxima CAS, as every single step can be controlled and evaluated. However, existing R packages may give a sufficiently good approximation as well.

### Maxima code for least square approximation of mu_a ###

# load table 1 and least square approximation

# Table1:matrix(N,Regular,Irregular,Amenorrhea, p.periods);

# p.periods.1:log(p.periods+10^(-6));

# Table1.1:matrix(N,p.periods.1);

# a0:create_list(1,i,N);

# a1:N;

# a2:N^2;

# a3:N^3;

# a4:N^4;

# A:transpose(matrix(a4,a3,a2,a1,a0))$

# Aplus:invert(transpose(A).A).transpose(A);

# Aplus.p.periods.1; [x^4,x^3,x^2,x,1].%$ Reg:%;

# exp(Reg); FMR(x):=''%;

# FMR(x-0.7);

############################################################

### Maxima code for least square approximation of mu_b ###

# load table 2 and least square approximation

# Table2:matrix(N,Regular,Irregular,Amenorrhea, p.periods);

# p.age:create_list(Table2[2][i],i,1,29); p.age[1]:10^(-4)$ p.age[28]:1-10^(-6)$ p.age[29]:1-10^(-4)$

# log:float(log(p.age/(1-p.age)));

# age:Table2[1];

# age2:age^2$

# age3:age^3$

# age4:age^4;

# age0:create_list(1,i,age);

# M:transpose(matrix(age0,age,age2, age3, age4));

# PseudoM:invert(transpose(M).M).transpose(M);

# a:0$ b:0$ c:0$ d:0$ e:0$;

# pol(x):=a+b*x+c*x^2+d*x^3+e*x^4;

# pol(x);

# log(x/(1-x))=A; solve(%,x);

# f:exp(pol(x))/(1+exp(pol(x)));

############################################################

## load validation data

df.validation <- data.frame(df2)

df.validation<-subset(df.validation,df.validation$current.contraception=="No")

df.validation<-subset(df.validation,df.validation$current.hrt=="No")

df.validation<-subset(df.validation,df.validation$pregnant=="No")

df.validation<-subset(df.validation,df.validation$breastfeeding=="No")

## exclude women answering "No, they have never been regular." to the question: "Do you have regular menstruations?"

df.validation<-subset(df.validation,df.validation$ec3_wq3==1 | df.validation$ec3_wq3==3 | df.validation$ec3_wq3==4 | is.na(df.validation$ec3_wq3))

## RAS functions

#number of periods

df.validation$mu_period<-1-exp(-0.00047014*(df.validation$period-0.7)^4+0.00894*(df.validation$period-0.7)^3-0.03072*(df.validation$period-0.7)^2+0.0861*(df.validation$period-0.7)-2.31719)

#optional age modifier

df.validation$a20<-0

df.validation$a20<-ifelse(df.validation$ovaries=="yes, one ovary",1,0)

df.validation$a20<-ifelse(is.na(df.validation$ovaries),0,df.validation$a20)

df.validation$a21<-ifelse(df.validation$smoke=="current smoker",2,0)

#age

df.validation$mu_age<-0.00470858*(df.validation$age+df.validation$a20+df.validation$a21)^2 - 0.08662429*(df.validation$age+df.validation$a20+df.validation$a21) - 7.64677252

df.validation$mu_age<-exp(df.validation$mu_age)/(1+exp(df.validation$mu_age))

#union

df.validation$ovaries<-ifelse(is.na(df.validation$ovaries),0,df.validation$ovaries)

df.validation$mu_all<-ifelse(df.validation$ovaries==3,1,df.validation$mu_period+df.validation$mu_age-(df.validation$mu_period*df.validation$mu_age))

## 4. Financial support

**RHINE:** This work was supported by the Norwegian Research Council [Grant No. 214123], the Bergen Medical Research Foundation, the Western Norwegian Regional Health Authorities [Grant No. 911 892 and 911 631], the Norwegian Labour Inspection, the Norwegian Asthma and Allergy Association, The Faculty of Health of Aarhus University, [Grant No. 240008], The Wood Dust Foundation [Grant No. 444508795], The Danish Lung Association, the Swedish Heart and Lung Foundation, the Vårdal Foundation for Health Care Science and Allergy Research, the Swedish Council for Working Life and Social Research, the Bror Hjerpstedt Foundation, the Swedish Asthma and Allergy Association, the Icelandic Research Council, and the Estonian Science Foundation [Grant No. 4350].

**Financial Support for ECRHS III:** Estonia: Tartu [Grant No. SF0180060s09] from the Estonian Ministry of Education. France: Ministère de la Santé. Programme Hospitalier de Recherche Clinique national 2010 ; Bordeaux: INSERM U897, Université Bordeaux Segalen; Grenoble: Comitée Scientifique AGIRadom 2011; Paris: Agence Nationale de la Santé, Région Île de France, domaine d’intérêt majeur; Germany: Erfurt: German Research Foundation [Grant No. HE 3294/10-1] Hamburg: German Research Foundation [Grant No. MA 711/6-1 and NO 262/7-1] Iceland: Reykjavik, The Landspitali University Hospital Research Fund, University of Iceland Research Fund, ResMed Foundation, Vegagerðin (The Icelandic Road Administration) Norway: Norwegian Research Council [Grant No. 214123], Western Norway Regional Health Authorities [Grant No. 911631], Bergen Medical Research Foundation. Spain: Fondo de Investigación Sanitaria [Grant No. PS09/02457, PS09/00716 09/01511, PS09/02185 and PS09/03190], Servicio Andaluz de Salud , Sociedad Española de Neumología y Cirurgía Torácica [Grant No. SEPAR 1001/2010]; Sweden: All centers were funded by The Swedish Heart and Lung Foundation, The Swedish Asthma and Allergy Association, The Swedish Association against Lung and Heart Disease. Fondo de Investigación Sanitaria [Grant No. PS09/02457] Barcelona:Fondo de Investigación Sanitaria [Grant No. FIS PS09/00716] Galdakao: Fondo de Investigación Sanitaria [Grant No. FIS 09/01511] Huelva: Fondo de Investigación Sanitaria [Grant No. FIS PS09/02185] and Servicio Andaluz de Salud Oviedo: Fondo de Investigación Sanitaria [Grant No. FIS PS09/03190] Sweden: All centers were funded by The Swedish Heart and Lung Foundation, The Swedish Asthma and Allergy Association, The Swedish Association against Lung and Heart Disease. Swedish Research Council for health, working life and welfare (FORTE) Göteborg: Also received further funding from the Swedish Council for Working life and Social Research. Umeå also received funding from Västerbotten Country Council ALF grant. Switzerland: The Swiss National Science Foundation [Grant No. 33CSCO-134276/1, 33CSCO-108796, 3247BO-104283, 3247BO-104288, 3247BO-104284, 3247-065896, 3100-059302, 3200-052720, 3200-042532 and 4026-028099] The Federal office for forest, environment and landscape, The Federal Office of Public Health, The Federal Office of Roads and Transport, the canton’s government of Aargan, Basel-Stadt, Basel-Land, Geneva, Luzern, Ticino, Valais and Zürich, the Swiss Lung League, the canton's Lung League of Basel Stadt/ Basel, Landschaft, Geneva, Ticino, Valais and Zürich, SUVA, Freiwillige Akademische Gesellschaft, UBS Wealth Foundation, Talecris Biotherapeutics GmbH, Abbott Diagnostics, European Commission [Grant No. 018996] (GABRIEL), Wellcome Trust [Grant No. WT084703MA]
