# Supplementary material for: Describing the status of reproductive ageing simply and precisely: A reproductive ageing score based on three questions and validated with hormone levels
Source: PLoS One. 2020 Jun 30;15(6):e0235478. doi: 10.1371/journal.pone.0235478 (PMC7326235; doi:10.1371/journal.pone.0235478)
Supplement: Attachment — Submitted filename: Rebuttal letter.pdf [file pone.0235478.s002.pdf]

# Rebuttal letter

Thank you very much for the opportunity to submit a revised version of the manuscript. Please find point-to-point responses to the reviewer's comments below. We thank the editor and the reviewers for valuable input and hope that you will find the responses acceptable. We have adapted the manuscript to the format requirement of PLoS ONE and the line numbers below, correspond to the revised manuscript with tracked changes.

## Reviewer #1:

**Comment 1:** The authors aim to generate an objective score for menopausal status. Overall, I'm still struggling with the utility of this score. It essentially uses features of the diagnostic criteria for menopause to give a score indicating the likelihood of menopause. However, the clinical features presented in table 3, suggest that it probably doesn't remove much heterogeneity to the diagnosis from the use of standard assessments or just asking the relevant information. I appreciate it doesn't claim to have predictive capability, merely being descriptive of current menopausal status, but is being validated against diagnostic criteria which have the flaws that the authors are trying to avoid. However, I'm unclear outside of the extremes of the range what knowing the score adds. I appreciate the example in research provided to try to demonstrate the utility in a continuous manner, but given that the validation step is arbitrary (and suspect that any number of similar scores using the information that form the diagnostic criteria for menopause could perform similarly), I'm not sure how useful score would be for clinical practice nor how valid it is as an intermediary value for research. Overall, I'm supportive of the concept of trying to make an objective diagnosis of menopausal status but remain skeptical of the score itself. In any case, its technically fine, so it will be up to the community to see if the use of this score takes off.

**Response 1:** We thank Reviewer #1 very much for appreciating the technical soundness of our reproductive aging score (RAS). We want to emphasize that the RAS is neither a probability measure nor diagnosing menopause, but as Reviewer #2 states, it is addressing: *"...a frequent issue when analyzing epidemiological data dealing with age at menopause."*

We describe the current status of reproductive ageing and we do not argue that our score will be necessarily more useful for clinical praxis, than the *Stages of Reproductive Ageing Workshop* classification (STRAW). However, the STRAW classification is flawed for epidemiological purposes due to the few categories, and because it relies on recalling the date of the last menstruation after one year, which is often challenging.

We reason that our score is precisely assessing the status of reproductive aging for epidemiological purposes or for clinical trials of medications that are time/ageing sensitive (such as hormone replacement therapy). If, for instance, women were more vulnerable to a certain health condition during a narrow time window within the menopausal transition, using the RAS as predictor in a regression analysis would reveal the corresponding signal.

The validation steps, mentioned by Reviewer #1 as arbitrary, are used for pedagogic purposes as most readers might be used to the established categorical definitions. Additionally, to our best knowledge there are no similar scores available and there is no other cohort providing questionnaire and hormone data combined with the possibility to elaborate and thoroughly validate such a score within a parallel cohort, as we have done.

We understand that clarity in certain passages of the manuscript could be improved, therefore we have modified the manuscript in order to address the reviewers concerns and elucidate the usefulness of the RAS:

**Title:** "Describing the ~~Defining menopausal~~ status of reproductive ageing simply and precisely: A reproductive ageing score ~~for epidemiologists, clinicians and the general public,~~ based on three questions and validated with hormone levels" (Lines 1 – 4)

**Abstract:** "…, that could give a simple and yet precise ~~measure~~ description of the current ~~menopausal~~ status of reproductive ageing, without hormone measurements, to be used by ~~women,~~ health professionals and researchers." (Lines 37 – 38)

"The RAS provides a useful and valid tool for ~~defining~~ describing the current status of reproductive ageing accurately,…" (Lines 53 – 54)

"This is useful for epidemiological research ~~medical practice~~ and ~~for women themselves~~ clinical trials." (Line 57)

**Introduction:** "The aim of the current paper is to mathematically describe the describe the current status of ~~menopausal transition as part of~~ reproductive ageing..." (Line 78)

**Discussion:** "The score was able to quantify degrees of the menopausal transition, which has so far not been possible, resulting in women ~~with various degrees of menopausal transition~~ at different stages of reproductive ageing being defined as one heterogeneous group." (Lines 284 – 287)

"This tool has great potential to offer new insights into health and disease during the perimenopausal phase, such as revealing the corresponding signal, if for instance, women were vulnerable to a certain health condition during a narrow time window within the perimenopausal phase." (Lines 287 – 290)

"These limitations are however also acknowledged in the STRAW +10 model [26], which, today, is considered to be the gold standard for ~~defining menopausal status~~ assessing reproductive ageing." (Lines 313 – 315)

**Conclusion:** "The RAS provides a new, innovative and useful tool ~~for defining menopausal status~~ to describe the current status of reproductive ageing accurately, on a continuous scale from 0.00 to 1.00, based on simple questions and without a need for blood measurements. … It thus is useful for epidemiological research and clinical trials of e.g. hormone replacement therapy ~~medical practice and for women themselves~~." (Lines 321 – 327)

**Comment 2:** It might be useful to add more detailed information about interpretation of the score beyond high and low, as in what is the conversion from RAS to the chance of menopause. Can the RAS be converted into a probability of menopause or is that what it already is supposed to denote i.e. does 0.86 mean an 86% chance of menopause? If so, this can be clarified for the reader.

**Response 2:** The RAS displays the current status of reproductive aging, rather than a probability/chance of menopause. Accordingly, only women with a score of 1.00 can be considered postmenopausal and only women with a score of 0.00 can be considered nonmenopausal. A score of 0.86 for example would have to be interpreted as not yet being menopausal but having completed 86% of the menopausal transition.

We now have added more detailed information about interpretation of the score to the manuscript:

"The RAS can be interpreted as an indicator of the progress of reproductive aging. Women with a score of 0.00 can be considered premenopausal and women with a score of 1.00 can be considered postmenopausal, while the intermediate values can be considered advancing degrees of reproductive aging in terms of decreasing fertility, respectively depletion of the ovarian reserve." (Lines 275 – 279)

**Comment 3:** Why was the effect of smoking and oophorectomy not evaluated from the same dataset, but rather an arbitrary value from a different set just added? Were other potential modifiers assessed? Previous history of anovulatory disorders eg PCOS?

**Response 3:** Evaluating determinants of age at menopause craves comprehensive analyses, as outlined in the cited articles, which would have been far beyond the scope of the current manuscript. However, the effect of smoking in the cited source (Dratva et al, N=5,288) is being evaluated in a population, which includes the cohort we use in the current analyses. Considering univariate oophorectomy, our data contained only few cases, therefore an evaluation within our population might be biased and we decided to implement the value determined by Bjelland et al in the HUNT study using 23,580 women.

**Comment 4:** Line 131- Perhaps shortening of cycles in the lead up to menopausal transition might also contribute to this? Reassuring to see very short cycles in table 1 increase the score.

**Response 4:** We agree and shortening cycles are considered by the RAS. This has been clarified in the manuscript.

"For women who report more than twelve menstruations per year it is expected to decline again, as shortening as well as lengthening cycles are an indicator of the beginning menopausal transition." (Lines 139 – 142)

**Comment 5:** Line 169- despite the aim to move away from the arbitrary categorizations, the cut offs used to validate the score also seem like consensus categorizations without the possibility of nuance. The FSH threshold of >20 seemed to be one that indicated anovulation and need for contraception rather than nonmenopause per se from the reference. A lower level would identify a more pure non-menopausal group.

**Response 5:** We assume the reviewer means the threshold "FSH ≤20IU/L" rather than "FSH threshold of >20". We mostly agree with the comment. However, given the already very good performance of the RAS to discriminate nonmenopausal women in the ROC analysis, the use of a lower threshold would lead to very minor differences and considering the cut-offs of Speroff et al are widely used, we decided to implement them in the current analysis. Please also see comment on categories and validation in Response 1.

**Comment 6:** Furthermore, FSH levels are known to fluctuate- was there possibility of including repeated values over a threshold? The ESHRE guidance for POI, uses two measurements of 25 on 2 occasions at least 4 weeks apart to diagnosis POI. An FSH of 15 would be considered high in the absence of raised LH, E2 which could suggest periovulatory value. Similarly not all women will have such a high FSH >80 post-menopause and gonadotropins can fall again after a number of years following menopause also.

**Response 6:** Fluctuations of FSH and LH are one of the reasons why threshold levels are indicators, rather than absolute diagnostic criteria of menopausal status. It is however commonly accepted and biologically plausible that elevated levels of FSH and LH over time indicate progressing reproductive aging. Unfortunately, repeated measurements were not available, as it is often the case in large population-based cohorts. The development of a score such as our RAS, however, requires such large studies, which also likely cover the whole range of FSH values throughout the menopausal transition. Furthermore, we could take advantage of a unique time window, as the median age of the women in the used cohort is very close to the average age of menopause. This means that the whole of the included population is in the relevant age range (38-66 years) and no extreme groups (as e.g. the elderly with possibly reduced Gonadotropins) distort the picture.

**Comment 7:** Can you plot scatterplots of the RAS by the three categorizations non, peri and post to visualize the performance of the score?

**Response 7:** Since all data points would be plotted on the same 3 vertical lines, we present a boxplot as our response, which also provides important additional information on medians (thicker black lines) and percentiles (see plot caption).

The plot below with menopausal categories (defined by the hormonal cut-offs used for the ROC analyses) shows that the median RAS is very low for nonmenopausal women, in the middle of the range for perimenopausal women, and very high for menopausal women. It also shows some disagreement (outliers) between the two classifications, mainly in the postmenopausal category. This implies that for some women hormone measurements, likely due to fluctuating levels, indicate a different status of reproductive ageing than age and number of periods.

We have now added this plot to the supporting information file, referring to it in the revised manuscript.

Manuscript: "Additionally, to visualize the performance of the RAS, we include a boxplot of the RAS against three commonly used categories defined by hormonal measurements in the supporting information." (Lines 192 – 194)

Supporting information: To visualize its performance, we plotted the reproductive ageing score against three commonly used categories defined by hormonal measurements as nonmenopausal with FSH ≤20IU/L and 17β-estradiol ≥147pmol/L; perimenopausal with FSH from 20IU/L to 80IU/L and 17β estradiol from 73pmol/L to 147pmol/L; and postmenopausal with FSH ≥80IU/L and 17β-estradiol ≤73pmol/L. (Lines 10 – 20, S1 Appendix)

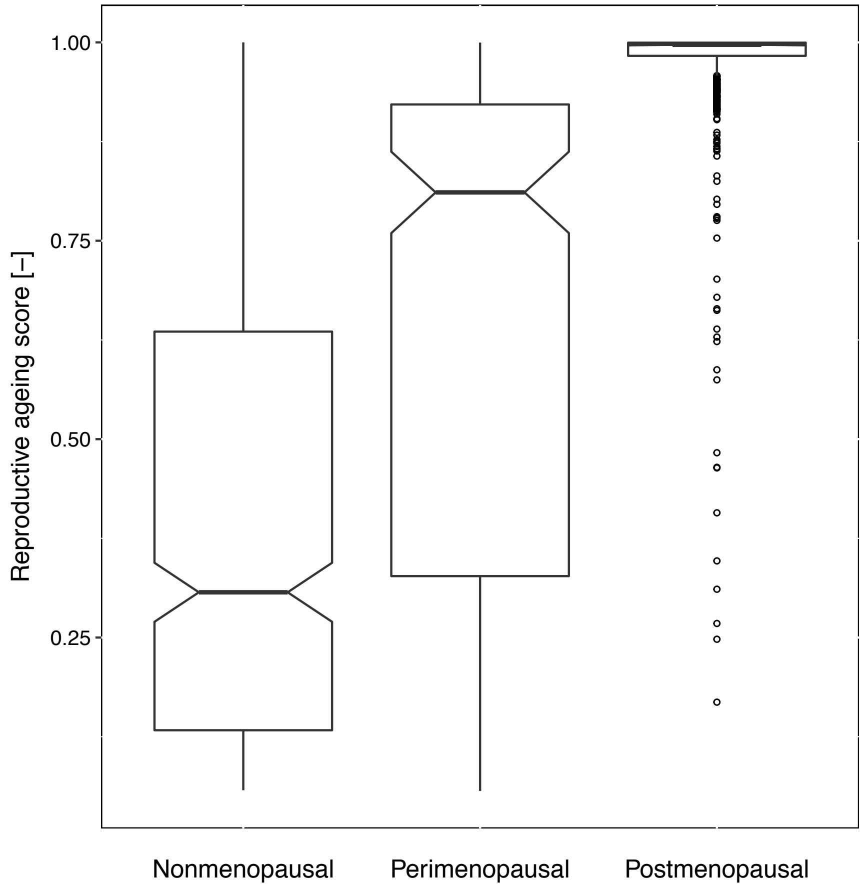


S1 Fig. **Boxplot of the reproductive ageing score by commonly used categories, defined by hormonal measurements;** The median is displayed as horizontal line within the boxes. The lower and upper hinges correspond to the 25th and 75th percentile. The upper and lower whiskers extend from the hinge to the largest, respectively smallest value, yet no further than 1.5 interquartile ranges. Data beyond the end of the whiskers are plotted individually.

**Comment 8:** As it was a longitudinal data set, was it possible to assess 'change in the number of periods per year over more than one year' as a factor?

**Response 8:** Due to the long follow-up time this could unfortunately not be evaluated.

**Comment 9:** Table 3- the score seems similar until a score more than 0.75 is reached. It seems that just asking how many periods in the last year would perform just as well as the RAS. And for a more subtle / nuanced assessment where there is not a clear-cut diagnosis then a combination of inhibin B, AMH, E2, FSH, LH, would be more predictive?

**Response 9:** We disagree with the first statement of this comment and want to emphasize again that the main use of the score is for epidemiological purposes on comprehensive datasets rather than daily clinical practice. The idea is not to calculate or predict a probability of menopause, but to describe the progress of reproductive aging during the menopausal transition. In Table 3 this is illustrated by the increasing standard deviations, which might be more informative than the mean number of periods. A combination of inhibin B, AMH, E2, FSH and LH would be very costly and not prioritized in e.g. countries with more pressing health matters, further it would be unlikely to be available for epidemiological purposes in large cohorts and most importantly: the need for such a large battery of expensive hormonal sampling would fall away if, as we believe, one can obtain similar or superior knowledge of a woman´s reproductive aging status just by asking a few simple inexpensive questions.

**Comment 10:** Can the methods add a paragraph or two explaining the concepts behind fuzzy mathematics for the non-expert reader?

**Response 10:** This has now been added to the manuscript.

"To develop the score we used techniques of fuzzy set theory, a mathematical concept to depict the biology of physiological processes [14, 15]. It was created in 1964 and successfully implemented in biology, artificial intelligence, and linguistics [14, 16, 17]. Unlike conventional mathematics, which does not allow vague expressions and demands that an object either is a member of a set or not, fuzzy sets are defined by a function (μ) assigning a value between *0.00* and *1.00* to an observation, representing the degree of belonging to a fuzzy set. The value *1.00* means that an object completely belongs to the set and the value *0.00* means the object does not at all belong to the set." (Lines 115 – 122)

## Reviewer #2:

The authors address a frequent issue when analyzing epidemiological data dealing with age at menopause. The idea of a continuous quantitative score, the RAS, based of simple measurements in any field study is attractive. Age at Menopause has indeed an indirect retrospective definition. The cessation of menses for more than 12 months does not always imply menopause and both FSH and Estradiol level are just mimicking what the gynecologist can observe directly.

Major Comment:

**Comment 11:** The development of the composite score needs to be more carefully explained. Although the calculation of mu_A and mu_B seem easy to understand, allowing to replicate the figures in the last column in table 1 and 2, the merging of the two indices is very briefly noted as obvious like the probability of the union of two sets with a non-zero interception. There is one basic assumption which may be obvious for the authors but absolutely not for the reader. My understanding is that for each year of age, the authors model the fraction of the women who were not regularly menstruating x times over the last 12 months.The later information is derived from all the women, whatever their ages are.

**Response 11:** Thank you very much for recognizing the merit of our developed score. We now explain the merging of the composite score in more detail.

"Subsequently, the value for the function $\mu_B$ is defined from $P(m_{age})$ and the RAS can be calculated as the union of $\mu_A$ and $\mu_B$ by adding the two sets ($\mu_A + \mu_B$) and subtracting the overlap, respectively the repeated elements of the intersection ($\mu_A \mu_B$): …

The union of $\mu_A$ and $\mu_B$ can be imagined within a three-dimensional Cartesian coordinate system, where $\mu_A$ is projected on the planes parallel to the one spanned by the x- and z-axis for each increment of $\mu_B$ and $\mu_B$ is projected on the planes parallel to the one spanned by the y- and z-axis for each increment of $\mu_A$. Subsequently the function, representing the union of $\mu_A$ and $\mu_B$ is formed by the maximum value of either function ($\mu_A$ or $\mu_B$) for any given coordinates of the plane spanned by the x- and y-axis." (Lines 165 – 175)

Minor comments:

**Comment 12:** The authors propose a correction for smoking and unilateral oophorectomy based on 2 publications. It has the merit of simplicity, but it could have been compared with models integrating these two covariates into the modeling of the log(mu_A) or the logit(mu_B)

**Response 12:** We agree in principle that the correction for smoking and unilateral oophorectomy could have been included into the modeling of $\mu_A$ and $\mu_B$. We chose not to do so, as including one or in this case even two co-variates would have immensely complicated the calculations and enlarged the presented formulae, likely without considerable changes of the final score. We believe that the current calculations are, as the referee points out "(*the calculation of $\mu_A$ and $\mu_B$ seem) easy to understand*", and simplicity was a very high priority

for the score development in order to inspire confidence in users without a mathematical background. Please also see our response to Comment 3.

**Comment 13:** The referee understands that the shape on figure 2 dictated a transformation that could cope with the strong nonlinearity with the constraints of staying within the range (0,1). Polynomial regression on the number of periods over the last year was an option, highly dependent on the data available in the range 12+, which are the less precise ones or may have different significances according to the woman's age, although they profoundly influence the highest degree of the selected polynomes. Did the authors try to compare their model with other models? Some discussions could be added.

**Response 13:** Throughout the development process of mu_A we considered a range of different degree polynomes and fitting criteria. We used the least squares method, as the variables are dependent and the Gauss-Markov theorem is applicable, which makes the least squares approximation the best linear unbiased estimator. In addition, the mean square deviation of the calculated functions reaches values very close to zero. This implies that there is a strong nonlinear correlation between the independent variables (number of periods in the last year and age) and the proportions, calculated within the development population. Since in the final score $\mu_A$ is strongly dependent on $\mu_B$, we further argue that the possibly less precise data (12+ menses in the last year) is largely being adjusted for. We have now addressed this in the Discussion section.

"To approximate the functions $\mu_A$ and $\mu_B$ we selected the least squares method, as the variables are dependent and the errors in the regression model are uncorrelated and have equal variances (Gauss-Markov theorem). In addition, the mean square deviation of the calculated functions reaches values very close to zero, implying a strong nonlinear correlation between the independent variables (number of periods in the last year and age) and the proportions, calculated within the development population. Concerning $\mu_A$, possibly less precise data in the upper range of menstruations per year (>12) is largely being adjusted for by the relation with age ($\mu_B$) after forming the final function (RAS)." (Lines 290 – 297)

**Comment 14:** The study largely benefits of two large, partly overlapping sets of data from mainly Caucasian women from the northern part of Europe. The authors indicate the exclusion of women either using contraceptive hormones, or being pregnant, or breastfeeding. How many women were exposed to intermittent progestin therapy, a common prescription at this period of life in European women? The two flowcharts seem to indicate that they have been also excluded?
Based on these two datasets, the validation study gives very good results at least for Caucasian women as correctly pointed out by the authors. The appropriate use of the proposed RSA assumes the availability of similar datasets corresponding to different homogeneous groups of women. If m_B data appear rather easy to get by simple questionnaires, m_A data may be more difficult to obtain.

**Response 14:** As the referee assumes correctly, women using intermittent progestin therapy have been excluded as well. This is now specified in the manuscript.

“We further excluded women from both populations who currently used exogenous sex hormones like contraceptives or hormone replacement therapy (including intermittent progestin therapy), …” (Line 104)

**Comment 15:** The final example on the association between the RSA and the Odds ratio of developing new- onset asthma brings additional value to the proposed score. It is worth noting the large confidence intervals in the late post-menopausal on the figure displaying the results with the traditional approach as compared with the ones on the predicted probability of new-onset asthma in relation to RAS. The comparison is tricky as the abscissa and the ordinates concern different entities and the regression methods is totally different. The shape of the confidence envelope partly reflects the complex nonlinearity of the transformation, but the rather "thin" right side remains intriguing. How was the confidence interval calculated?

**Response 15:** The confidence interval was calculated on the RAS scale and then mapped to the response scale by first producing predictions on the RAS scale and subsequently the standard errors of the predictions, giving the confidence interval of the RAS as:

upper bound= prediction +(1.96 * standard error)

lower bound= prediction - (1.96 * standard error)
