# Supplementary material for: Describing the status of reproductive ageing simply and precisely: A reproductive ageing score based on three questions and validated with hormone levels
Source: PLoS One. 2020 Jun 30;15(6):e0235478. doi: 10.1371/journal.pone.0235478 (PMC7326235; doi:10.1371/journal.pone.0235478)
Supplement: Attachment — Submitted filename: 20200604_Rebuttal letter 2.pdf [file pone.0235478.s003.pdf]

Rebuttal letter #2

Thank you very much for the opportunity to submit another revised version of the manuscript. Please find point-to-point responses to the reviewer's comments below. We thank the editor and the reviewers for input that clearly added value to the manuscript, and we agree to the publication of the review history. The line numbers below correspond to the revised manuscript with track changes.

Red font: First revision

Purple font: Second revision

Reviewer #1:

We thank Reviewer #1 very much for valuable comments and suggestions.

Reviewer #2:

The revised version addresses most of the comments. However the following suggestions could be taken into account to improve the readability and the reproducibility of the manuscript

Reviewers' major comment: Although the revised version adds substantial technical information, it does not fully address the basic assumption made by the authors which implies that the probability of either not regularly menstruating or being without menses according to the number of periods within the last 12 months (function mu_A(Period)) does not depend on the woman's age, allowing to calculate for each year a probability of being either amenorrheic or not regularly menstruating by using the classical formula for the probability of the union of two sets with non null intersection. This is an important assumption which should be made more clear for the reader.

Authors' response: Please excuse us for not addressing this comment in the way it was intended. We now have added this important assumption to the manuscript:

"The calculated probability of being not regularly menstruating or without menses, according to the number of periods within the last twelve months (μ_A), does not depend on the woman's age, thus allowing to calculate the probability for each year of being either amenorrheic or not regularly menstruating. Thus ~~and~~ the RAS can be calculated as the union of μ_A and μ_B by adding the two sets ($\mu_A + \mu_B$) and subtracting the overlap, i.e. the repeated elements of the intersection ($\mu_A\mu_B$):" (Lines 167 - 172)

Minor comments:

Reviewers' comment 12: The authors propose a correction for smoking and unilateral

oophorectomy based on 2 publications. It has the merit of simplicity, but it could have been compared with models integrating these two covariates into the modeling of the log(mu_A) or the logit(mu_B)

Reviewers' comment on response 12: We agree with the authors that the proposed score should be easy to calculate justifying their rather simple proposal, but it reincorporates a rather crude categorization when the authors try to convince the reader to use a continuous variable for quantifying the reproductive aging status.

Authors' response: Thank you for your comment. Our chosen approach leaves the user at liberty to decide in what way smoking behavior should be entered into the model. In case the user has access to a database where smoking behavior is precisely characterized, he or she may easily choose to use the RAS without smoking adjustment and adjust for this variable in a subsequent (regression) analysis. Similarly, the user may choose to evaluate the effects of unilateral oophorectomy by other means, such as sex hormone concentrations.

To clarify, we made the following changes to the manuscript:

"Additionally, two optional modifiers were introduced to μ_B:" (Line 158)

"In case that it is desirable to evaluate smoking behavior and/or unilateral oophorectomy separately or in a different manner, these modifiers may be removed from the calculation of the RAS." (Lines 307 - 309)

Reviewers' comment 13: The referee understands that the shape on figure 2 dictated a transformation that could cope with the strong non linearity with the constraints of staying within the range (0,1). Polynomial regression on the number of periods over the last year was an option, highly dependent on the data available in the range 12+, which are the less precise ones or may have different significances according tho the woman's age, although they profoundly influence the highest degree of the selected polynomes. Did the authors try to compare their model with other models? Some discussions could be added.
Comment on the response 13: the referee tried to replicate the observed results using the two tables provided in the manuscript. If the logit regression for the age- dependent frequencies gives rather similar results, but not exactly the same ones, for adjusting and graphing the mu_B curve, the adjustment of the mu_A part remains less straightforward due to the right part of the curve, i.e. number of periods per year above 12. Various methods and selection of the non- linear transform could lead to rather good adjustments. The justification for the least square method as well the reference to the Gaussian Markov theorem has not evident theoretical basis for a regression which concerns either integer values or proportion, although it gives satisfactory results from the practical point of view. My suggestion is to suppress the corresponding sentence without entering into too much irrelevant detail.

Authors' response 13: We agree with the referee and have removed the respective sentence. (Lines 295 – 297)

Reviewers' comment 14: The study largely benefits of two large, partly overlapping sets of data from mainly caucasian women from the northern part of Europe. The authors indicate the exclusion of women either using contraceptive hormones, or being pregnant, or breastfeeding. How many women were exposed to intermittent progestin therapy, a common prescription at this period of life in European women? The two flowcharts seem to indicate that they have been also excluded?

Reviewers' response 14: OK for the added sentence

Reviewers' comment 14 bis: Based on the two large available datasets, the validation study gives very good results at least for caucasian women as correctly pointed out by the authors. The appropriate use of the proposed RAS assumes the availability of similar datasets corresponding to different homogeneous groups of women. If m_B data appear rather easy to get by simple questionnaires, m_A data may be more difficult to obtain.

Reviewers' comment on the absence of response to comment 14 bis: It is suggested to add in the supplementary material the R- code for adjusting the datasets, in order to allow epidemiologists to replicate the process in other settings. As an additional comment, it was not clear for the reader how the authors used the Maxima CAS software considering the absence of real theoretical development. A clear statement should be made about the only necessity of the R statistical package to replicate the results.

Authors' response 14 bis: We have now added the R code for adjusting the datasets to the supplementary material.
We used the Maxima CAS software for the least square approximation. Maxima CAS is an open-source software and can be downloaded here:
https://sourceforge.net/projects/maxima/files/
Maxima CAS is a specialized computer algebra system that yields high precision numerical results by using exact fractions, arbitrary-precision integers and variable-precision floating-point numbers. It is further capable to include and compare differentiation, integration, Taylor series, Laplace transforms, ordinary differential equations, systems of linear equations, polynomials, sets, lists, vectors, matrices and tensors. To replicate the current calculations, we recommend using Maxima CAS, as every single step can be easily evaluated. We acknowledge that existing R packages provide a viable alternative.

~~All calculations~~ The least square approximations to develop the RAS were performed using the Maxima CAS (Computer Algebra System) software [23]. All other calculations ~~to validate~~ including the validation of the RAS were performed using the R statistical package [24]. (Lines 200 – 202)

Another strength is that the RAS can be easily ~~reproduced and implemented~~ used with ~~in~~ all common~~ly used~~ statistical software and spread sheets by applying the final formula (Equation 7) to a dataset. To replicate the development of the RAS (least square approximations) in other settings we recommend using the open-source software Maxima CAS (https://sourceforge.net/projects/maxima/files/), a specialized computer algebra system that yields high precision numerical results and is capable of including and evaluating complex operations, such as Taylor series and Laplace transforms as well as linear algebra tools like matrix operations, which have been used for the current calculations. (Lines 310 – 317)

Reviewers' comment 15: The final example on the association between the RAS and the Odds ratio of developing new- onset asthma brings additional value to the proposed score. It is worth noting the large confidence intervals in the late post-menopausal on the figure displaying the results with the traditional approach as compared with the ones on the predicted probability of new- onset asthma in relation to RAS. The comparison is tricky as the abscissa and the ordinates concern different entities and the regression methods is totally different. The shape of the confidence envelope partly reflects the complex non linearity of the transformation, but the rather "thin" right side remains intriguing. How was the confidence interval calculated?

Reviewers' comment on the response 15: The response remains rather vague for the reader. I understand that the authors performed in the previous published analysis a classical regression analysis of the occurrence of asthma episodes in relation to the menopausal status and perhaps age and here they are performing a second one of the same variable in relation to RAS and perhaps age. I assume that the second one was at least quadratic on RAS to take into account a possible more pronounced effect during the peri- menopausal period. Subsequently, I assume that the authors used some sort of delta method to calculate the confidence interval of the predicted probability from the variance of the regression variable taking into account the var/cov matrix of the adjusted coefficients of the model. How it ends up with a very thin punctual confidence interval when RAS is 1 is not totally obvious. It could be useful to add more details in the appendix.

Authors' response 15: We regret misreading the initial comment and providing the confidence interval calculations only for the regression coefficient. To calculate the confidence band depicted in the S3 Fig. we used the predict.loess() function (locally estimated scatterplot smoothing) with the formula 'y ~ x', which uses a t-based approximation. Detailed documentation can be found here:
https://www.rdocumentation.org/packages/stats/versions/3.6.2/topics/predict.loess
We are aware that it might be argued here to use a GAM 'general additive model' function with a formula, such as e.g. y ~ s(x, bs = "cs"), which would give a slightly differently shaped confidence band.
However, while the presented example on asthma and reproductive aging constitutes a very interesting and relevant subject, we included it in the current paper solely as an application of the RAS. Upon publication of the RAS we plan to use the RAS for in-depth analyses on associations between respiratory health and reproductive aging.

To clarify, we modified the caption of the respective figure accordingly:

S3 Fig. Probability of new-onset asthma by reproductive aging score for 2085 women, incl. 95% confidence ~~interval~~ band, calculated using ~~(~~t-based locally ~~weighted~~ estimated scatterplot smoothing~~)~~. (Lines 49 – 50 of S1 Appendix)
